# Effects of Job Crafting and Leisure Crafting on Nurses' Burnout: A Machine Learning-Based Prediction Analysis

**DOI:** 10.1155/2024/9428519

**Published:** 2024-06-20

**Authors:** Yu-Fang Guo, Si-Jia Wang, Virginia Plummer, Yun Du, Tian-Ping Song, Ning Wang

**Affiliations:** ^1^ School of Nursing and Rehabilitation Shandong University, Jinan, Shandong, China; ^2^ Institute of Health and Wellbeing Federation University Australia, Victoria, Australia; ^3^ Qilu Hospital of Shandong University Dezhou Hospital, Dezhou, Shandong, China

## Abstract

**Aim:**

To explore the status of job crafting, leisure crafting, and burnout among nurses and to examine the impact of job crafting and leisure crafting variations on burnout using machine learning-based models.

**Background:**

The prevalence of burnout among nurses poses a severe risk to their job performance, quality of healthcare, and the cohesiveness of nurse teams. Numerous studies have explored factors influencing nurse burnout; however, few involved job crafting and leisure crafting synchronously and elucidated the effect differences of the two crafting behaviors on nurse burnout.

**Methods:**

Multicentre cross-sectional survey study. Nurses (*n* = 1235) from four Chinese tertiary hospitals were included. The Maslach Burnout Inventory-General Survey, the Job Crafting Scale, and the Leisure Crafting Scale were employed for data collection. Four machine learning algorithms (logistic regression model, support vector machine, random forest, and gradient boosting tree) were used to analyze the data.

**Results:**

Nurses experienced mild to moderate levels of burnout and moderate to high levels of job crafting and leisure crafting. The AUC (in full) for the four models was from 0.809 to 0.821, among which the gradient boosting tree performed best, with 0.821 AUC, 0.739 accuracy, 0.470 sensitivity, 0.919 specificity, and 0.161 Brier. All models showed that job crafting was the most important predictor for burnout, while leisure crafting was identified as the second important predictor for burnout in the random forest model and gradient boosting tree model.

**Conclusion:**

Even if nurses experienced mild to moderate burnout, nurse managers should develop efficient interventions to reduce nurse burnout. Job crafting and leisure crafting may be beneficial preventative strategies against burnout among nurses at present. *Implications for Nursing Management*. Job and leisure crafting were identified as effective methods to reduce nurse burnout. Nurse managers should provide more opportunities for nurses' job crafting and encourage nurses crafting at their leisure time.

## 1. Background

Nurses constitute the largest proportion of the healthcare workforce worldwide and play an essential role in clinical treatment, illness prevention, and health promotion. However, because of extensive job demands, scarce resources, work-family conflicts, and complex clinical work environments, nurses experience several burnout symptoms, such as emotional exhaustion, cynicism, and reduced professional efficacy [1, 2]. These phenomena adversely affect the quality of care, patient safety and satisfaction, job performance, turnover rate, and the physical and mental health of nurses [3, 4]. According to a nationwide survey conducted in the United States of America, 16.6%–30.0% of nurses (3,957,661 samples) reported experiencing burnout and 31.5% of them listed burnout as a contributing factor to their decision to leave their positions [5]. A cross-sectional survey in 12 European countries showed that 10%–78% of nurses had burnout symptoms [6]. In the mainland China, the burnout rate is also high; for example, in Shanghai, it is 45.1% [7], and in Hunan, it is at 34.9% [8]. Given the current high levels of burnout among nurses worldwide, it has become critical for hospital administrators, nurse managers, and nurses themselves to gain a deeper understanding of the factors that contribute to burnout to enhance the quality of clinical care, stabilize nurse retention, and improve the physical and mental health of nurses.

Job crafting is defined as the actions employees take in modifying their tasks and work relationships to promote their cognitive understanding of the job meaning and attain a greater person-job fit [9]. Previous studies have highlighted that job crafting is not only beneficial to employees' health (e.g., well-being and general health) [10, 11], work attitude (e.g., job satisfaction and organizational commitment) [12], and behavior (e.g., work engagement and creativity) [13] but is also conducive to organizational performance (e.g., quality of care and group innovation) [14, 15]. Literature review shows that increasing research had investigated the relationship between job crafting and burnout in nurses. Cumulative evidence has shown that job crafting can maintain a proactive balance between job demands and resources by fostering self-driven work behavior, consequently alleviating nurse burnout [16]. Several studies have found a moderate-to-high influence of job crafting on burnout [16, 17], whereas others have reported a rather small effect of job crafting on burnout [18]. Because the effect sizes of these studies were inconsistent, further investigation is required to provide new evidence.

Leisure crafting is a novel strategy for dealing with depleted resources and was firstly defined by Petrou and Bakker [19]. This strategy refers to individuals' proactive and self-initiated pursuit of off-job life to fulfill their goals, interpersonal connections, learning, and personal growth. Unlike job crafting, leisure crafting motivates individuals' passions and satisfies their psychological requirements by shaping their leisure activities in a proactive, deliberate, and serious manner [20, 21]. Existing studies indicated that leisure crafting can reshape the task and relational bounds of individuals' leisure, improve their sense of mastery, and help them acquire resources [22, 23]. Leisure crafting is believed to address individuals' needs and values and improve their ability to handle workplace stressors, thereby averting the negative effects of job demands [24]. Few studies have explored the effects of leisure crafting on work-related attitudes and behaviors, particularly burnout issues. Moreover, little is known about whether job crafting and leisure crafting have similar effects on burnout.

Conservation of resources' theory assumes that individuals have the motivation to invest their resources for accumulating additional resources to protect their health and well-being [25]. Based on the conservation of resources' theory, an excessive workload is a persistent threat to nurses' valued resources, which results in burnout [26]. While nurses' accumulation of resources can sustain and protect additional resources, which greatly alleviate burnout among nurses, several studies supported that both job crafting and leisure crafting play vital roles in acquiring and adjusting resources [10, 27]. Job crafting and leisure crafting represent resource gain processes as nurses cope with stressful job demands and enrich their leisure time that motivate them and enhance their ability to perform well [28]. Resources gained from job crafting can improve nurses' ability to better fulfill their work obligations, which is conducive to reduce burnout [29]. Resources activated and replenished from leisure crafting can spill over to the work domain to improve nurses' work engagement and hence lighten nurse burnout [27]. Therefore, based on theoretical basis and the empirical studies, the study puts forward hypothesis 1: job crafting and leisure crafting are negatively influencing nurse burnout.

At present, most studies have investigated the factors influencing burnout using multiple linear regression models, which have high requirements for data features (including normal distribution and homoscedasticity). Machine learning, a common artificial intelligence-driven technology, has been successfully integrated into the field of health risk assessment. Unlike traditional statistics, machine learning models do not have any rules for data distribution and learn from provided samples to explore the complex and nonlinear relations among measured variables [30]. Several studies have applied machine learning to test predictors of burnout and achieved good findings. However, most of them only used one type of machine learning model (e.g., convolutional neural network, multitask learning technique, and decision tree) [31, 32], which does not avoid bias caused by the model and confirms the most significant predictors of burnout.

The main objectives of this study were to provide a comprehensive description of job crafting, leisure crafting, and burnout among nurses from Chinese tertiary hospitals. We also aimed to evaluate the effects of job crafting and leisure crafting on burnout by developing and validating four machine learning-based models.

## 2. Methods

### 2.1. Study Design

The cross-sectional multicenter study design was employed to describe the status of job crafting, leisure crafting, and burnout among nurses and explore the associations among these variables.

### 2.2. Participants

There are 107 tertiary hospitals in Shandong Province, China (https://www.doc88.com/p-11461558491027.html). A convenience sampling method was used to recruit hospitals from the 107 tertiary hospitals. Nurses from the included hospitals, who met the inclusion and exclusion criteria, were required to participate in the online survey. The inclusion criteria included registered and licensed practical nurses who directly cared for patients. Nurses on sick leave or those in the process of turnover were excluded from the study.

A total of 1,754 nurses from the four tertiary hospitals were invited to complete the online questionnaires, and 1,235 responses were included in the final analysis (Supplementary file “Participation rate and sample size”).

### 2.3. Measures

A personal demographic form, the Maslach Burnout Inventory-General Survey (MBI-GS), the Job Crafting Scale (JCS), and the Leisure Crafting Scale (LCS) were used for the online survey.

The personal demographic form included 10 questions to investigate nurses' sociodemographic information, such as sex, age, years of service, academic degree, marital status, do they have a child/children, professional qualifications, specialty area, monthly income, and shift work.

The MBI-GS was used to assess the nurses' burnout status. This 16-item seven-Likert scale was developed by Maslach and Jackson [33]. The Chinese version was translated by Li and Shi [34]. Three dimensions of the MBI-GS are “emotional exhaustion,” “cynicism,” and “reduced professional efficacy.” A seven-point Likert scale (0 = never, 6 = every day) was used to score the items. The following equation, developed by Kalimo et al. [35], was used to calculate the total MBI-GS score. Total score for burnout = score for “emotional exhaustion” × 0.3 + score for “cynicism” × 0.3 + score for “reduced professional efficacy” × 0.4. The minimum and maximum scores for burnout range from zero to six, with higher scores indicating severe levels of burnout. Cronbach's alpha coefficients for the MBI-GS were 0.88 in the study.

The JCS used to measure job crafting was developed by Tims et al. [36]. The Chinese version's validity and reliability were tested by Liao [37]. This 21-item scale has four dimensions (increasing structural job resources, increasing social job resources, increasing challenging job demands, and reducing obstructive job demands) and scores on a five-point Likert scale. The score for job crafting is the average score of the total items. Higher scores indicated greater job crafting experienced by nurses. In this study, Cronbach's alpha coefficients for the JCS were 0.95.

The nine-item LCS, developed by Petrou and Bakker [19], measured nurses' crafting during their leisure time. The validity and reliability of the Chinese version were examined by Guo et al. [38]. A five-point Likert scale (1 = not at all, 5 = very many) was used to score each item. The LCS score is the average of nine items. The LCS had good validity and reliability, with Cronbach's alpha coefficients of 0.95 in the current study.

### 2.4. Data Collection

This study was conducted at four tertiary hospitals between June 3 and October 31, 2022. The Wenjuanxing-Enterprise edition (Changsha Ranxing Information Technology Co., Ltd., Changsha, China) was used to develop the online survey. Two research assistants were recruited from each hospital. All research assistants were given a three-hour investigation training session before conducting the survey. Research assistants delivered an online questionnaire link and explanatory statements to the nurses via e-mail, WeChat, and other communication apps. Nurses who met the inclusion criteria were asked to complete the survey. Two push notifications (Dear participants, please remember to complete the online survey. Thank you.) were sent to nurses. The survey took 10–15 minutes to complete.

### 2.5. Ethical Considerations

Ethical approval (Human Sciences Ethics Committee of School of ^*∗∗*^, one University No. 2020-R-030) and hospital permissions were obtained for this study. Nurses who submitted the online survey were considered to have provided informed consent.

### 2.6. Data Analyses

Data were analyzed using SPSS 24.0, with descriptive analysis, *t*-tests (to compare burnout scores between two groups), ANOVA (to compare burnout scores' differences among three or more groups), and correlation tests (to evaluate the correlations among the measured variables). Kurtosis and skewness were used to describe the normal distribution of measured variables. Values for kurtosis were from −0.237 to 1.002, and values for skewness were between −0.724 and 0.392, indicating that, generally, the data were normally distributed. According to the Harman single-factor analysis, 36.7% of the variance could be explained by one factor, which suggests that no significant common method variance was found in the study. K-means clustering analysis was used to divide burnout into a low burnout group and a high burnout group.

A computer-generated random number sequence divided the data into training (70%) and validation (30%) cohorts. Python 3.9 was employed to conduct four machine learning algorithms (logistic regression model, support vector machine, random forest, and gradient boosting tree) to obtain models for predicting nurse burnout. The logistic regression model is one kind of generalized linear regression categories. Support vector machine employs kernel functions to map linearly indivisible data to a multidimensional feature space, which could deal with complex data such as high dimensional, nonlinear, and small sample size. Random forest is an integrated algorithm which uses decision trees as the main classifier. Random forest is applied to issues of classification and regression. Gradient boosting tree includes decision tree and gradient boosting. It can deal with tasks of classification and regression via additive model and forward distribution algorithms.

The burnout prediction models included 16 variables (independent variables: job crafting and leisure crafting; covariates: significant demographic characteristics; dummy variables: eight variables). Five repetitions of the 10-fold cross-validation were conducted to optimize the model parameters. The area under the receiver operating characteristic curve (AUC), accuracy, sensitivity, specificity, and Brier were calculated to compare the predictive performance of the models. Brier represents the average-squared distance from the predicted probability of the model to the actual probability. The lower the Brier scores, the better the model performance. A two-sided *p* value ≤0.05 was considered statistically significant.

## 3. Results

### 3.1. Demographic Characteristics and Burnout of Nurses

Most nurses were women (95.8%), married (80.9%), held bachelor's degrees (85%), and had a child/children (76.2%). Over half were 30–39 years old, had temporary employment, had worked less than 11 years in medical and surgical departments, and had shift work more than four times per month. Of these, 46.4% were lead nurses, and only 23.3% of the nurses' income was higher than 9000 Yuan (1,231 $ USD) (Table 1).

The results of the bivariate statistical analysis showed that nurse burnout levels were significantly different according to age, years of service, marital status, child/children status, professional qualifications, specialty area, and shift work (each *p* < 0.01).

### 3.2. Descriptive and Correlation Analyses among Burnout, Job Crafting, and Leisure Crafting

The burnout level for nurses was 2.02 ± 1.05, indicating that nurses experienced mild-to-moderate levels of burnout. The average score for job crafting was 4.05 ± 0.56. The average score for leisure crafting was 3.84 ± 0.78, suggesting that nurses had moderate-to-high levels of job and leisure crafting (Table 2).

The correlation analysis showed that burnout was significantly negatively correlated with job and leisure crafting (each *p* < 0.01) (Table 2). Nurses who experienced high burnout generally engaged in less job and leisure crafting.

### 3.3. K-Means Clustering Analysis for Burnout

According to the K-means clustering analysis, two clusters for burnout were found: the low-burnout group (final cluster center 1.24, *n* = 678) and the high-burnout group (final cluster center 2.97, *n* = 557) (Table 3).

### 3.4. Model Performance

The binary logistic regression analysis showed that job crafting, age, medical department, paediatric department, and shift work significantly influenced nurse burnout. Nurses who had lower job crafting, were younger in age, worked in medical and paediatric departments, and shifted work had a higher risk of experiencing severe burnout. Supplementary Files eTable 1 and eFigure 1 present the regression model results.

Support vector machine (SVM), random forest, and gradient boosting tree were employed to evaluate significant factors influencing burnout. The importance of the permutation features was calculated using the three machine learning algorithms. In the SVM model, the top five predictors were job crafting, age, child/children status, years of service, and leisure crafting (Supplementary file eFigure 2). In the random forest and gradient boosting tree models, the top five predictors were job crafting, leisure crafting, age, years of service, and professional qualifications (Supplementary file eFigures 3 and 4).

**Figure 1:**
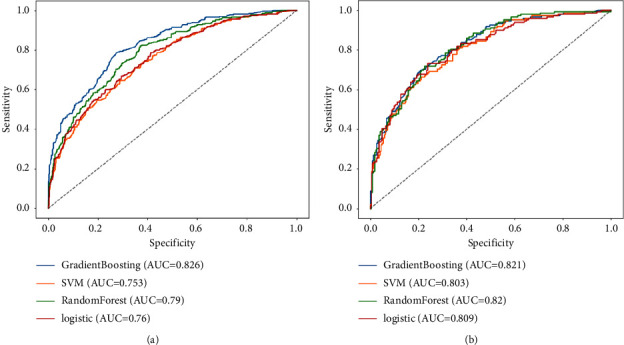
ROC curves of different machine learning models in (a) the training cohort and (b) the validation cohort.

The performance of each model is summarized in Table 4. The receiver operating characteristic (ROC) curves for the validation cohort are shown in Figures 1(a) and 1(b). The gradient boosting tree model performed the best, with an AUC of 0.821, accuracy of 0.739, sensitivity of 0.470, and specificity of 0.919. The Brier score for the gradient boosting tree was the lowest (0.161), indicating that the model was reliable.

## 4. Discussion

This study aimed to explore the status of job crafting, leisure crafting, and burnout among Chinese nurses and exam the effects of job crafting and leisure crafting on burnout using four machine learning algorithms. This study is one of the first investigations on this topic. We found that nurses experienced mild-to-moderate levels of burnout and moderate-to-high levels of job and leisure crafting. Furthermore, compared with leisure crafting, job crafting played a greater role in predicting burnout in SVM, random forest, and gradient boosting tree models. These important findings suggest that nurses plan their efforts to promote their job and leisure crafting and that nurse managers should adopt effective strategies to reduce burnout symptoms among nurses.

In the current study, nurses had mild-to-moderate levels of burnout. These results are supported by previous studies conducted in other countries [39, 40]. As nurses experience mild-to-moderate burnout, both nurse managers and nurses pay less attention to this chronic, persistent syndrome, which leads to severe outcomes in nurses' physical and mental health, job performance, and organizational commitment [41, 42]. Accordingly, raising managers' and nurses' concerns about burnout is vital, especially in mainland China. Furthermore, effective personal-oriented, organizational-oriented, and personal-organizational combined interventions should be implemented to reduce burnout among nurses.

In this study, the nurses experienced moderate-to-high levels of job crafting. This finding is consistent with the studies of Alharthi et al. [43] and Harbridge et al. [44]. A qualitative study revealed that nurses had passion and strengths in job crafting. They were actively job crafting in all aspects via activities, such as techniques' training, participating in working teams and committees, and being involved in programs [45]. Nurses proactively initiated and altered clinical tasks to address the requirements of their vulnerable patients, which could promote the quality of care and achieve more meaning in their jobs [46].

We found, quite interestingly, that nurses experienced moderate-to-high levels of leisure crafting. Studies have indicated that leisure crafting is an individual's adaptive behavior in leisure life, which can positively benefit work engagement, personal achievement, and organizational performance [47, 48]. Several leisure activities were believed to enhance individuals' leisure crafting, such as hobby participation, enjoying activities, and seeking a sense of purpose during leisure time [49]. Therefore, nurses are encouraged to pursue their hobbies and engage in leisure activities to produce crafting behaviors outside their work.

In the present study, four machine learning models were used to evaluate the effects of differences in job and leisure crafting on burnout among nurses. Although the predictive capacity of the models was less satisfactory (AUCs ranged from 0.803 to 0.821), they can help nurse burnout management and their prediction can be improved by including more influencing variables. The sensitivity of the four models ranged from 0.128 to 0.470, indicating a low capacity to target nurses at a high risk of burnout. A possible reason is that some important predictors were not included in the models; as a result, the models could not sensitively include nurses with burnout. The specificity of the four models (ranging from 0.919 to 0.995) was good in the study, indicating that the models specifically distinguished burnout from similar syndromes. Among the four models, the gradient boosting tree exhibited the best performance, with good sensitivity and calibration efficacy. The performance of the SVM model was similar to that of the random forest model, whereas that of the logistic model was far from satisfactory.

In this study, job crafting was the most important predictor of burnout in the four models. In contrast, the effect size of job crafting on burnout was small in the logistic regression analysis (odds ratio = 0.123). Martinez et al. [50] reported that job crafting can explain 15.7%–19.7% of the variance in burnout in four dimensions (personal impact dimension, job dissatisfaction, motivational abandonment, and social climate dimension) among male nurses. Roskova and Faragova [18] reported that job crafting combined with age and position explained 14% of the variability in burnout among full-time employees. According to the Job Demands-Resources model, overwhelming job demands and limited job resources lead to burnout and job crafting is one of the mediating factors in this relationship. High job crafting encourages individuals to reframe their perceptions of work, engage in more workplace relationships, and change the nature of their tasks, which could decrease burnout and increase job satisfaction and well-being [51]. Considering the mild but significant effect of job crafting on nurse burnout, nurse managers and nurses must foster nurses' crafting behaviors in their clinical work through several typical interventions (e.g., job crafting workshops and job crafting exercises) [52, 53].

In the study, we creatively revealed the predictive value of leisure crafting on burnout using four machine learning models. Leisure crafting was the second or fifth most important feature for burnout in the SVM, random forest, and gradient boosting tree models. However, leisure crafting was not included in the final logistic regression model. Studies have reported that off-job crafting enables individuals to learn new things, expand existing hobbies, and have new personal connections, which can help them overcome severe challenges, promote personal growth, and achieve a good balance between job and leisure [54, 55]. Ugwu [56] demonstrated that leisure crafting alleviates the negative effects of counterproductive work behaviors caused by high job demands. Therefore, nurse manager and nurses should be aware of the importance of leisure crafting in burnout. Various leisure activities should be organized in establishing new interpersonal relationships, developing new skills, and learning new meanings in work and life [57].

Despite the fact the correlations between job crafting, leisure crafting, and burnout had been tested in the study, several limitations in this present study need to be considered. First, data were collected from four Chinese tertiary hospitals. Data from other hospital levels were lacking, which might have influenced the performance of the model in the external dataset. We suggest collecting sufficient data from different hospitals to validate the model. Second, regarding the cross-sectional study design, the effects of job and leisure crafting on burnout did not present a cause-and-effect relationship. Therefore, a longitudinal study is needed to better understand the predictions of job and leisure crafting on nurse burnout. Third, the study focused only on limited characteristics of nurses, such as demographic, job crafting, and leisure crafting. This might be the primary reason for the low sensitivity of the models. Therefore, several significant factors influencing burnout (e.g., job stress, job resources, work-family conflict, collegial support, and leadership) should be investigated and analyzed in future studies. Fourth, 52 male nurses were included in the study. Generalization of the model for male nurses should be done with caution, and further surveys of this demographic are needed to clarify the model found in the study. Finally, nurses who were on sick leave and in the process of turnover were excluded from the study. Nurses in these conditions may experience high levels of burnout. Therefore, further studies could recruit nurses in these conditions to promote the generalization of the studies.

## 5. Conclusions

Burnout is a serious issue among nurses worldwide, raising great concerns among hospital administrators and nurse managers. In this study, nurses experienced mild-to-moderate levels of burnout; moreover, they had moderate-to-high levels of job and leisure crafting. These findings indicate that despite nurses having some burnout symptoms, they tend to apply crafting activities to increase their clinical skills and confidence and promote their interpersonal relationships and self-development. According to the machine learning-based predictive models, job crafting and leisure crafting were significant predictors of burnout, and job crafting was the most important predictor of burnout in the four models. Therefore, nurse managers are encouraged to create a casual crafting environment and take effective measures to improve nurses' crafting behaviors, ultimately reducing their burnout and promoting their clinical performance and organizational commitment.

## 6. Implications for Nursing Management

Burnout is a critical risk factor for the organizational health (e.g., organizational commitment and development) of hospitals and physical (e.g., inflammation, pain, and sleep disorder) and mental health (e.g., anxiety and depression) of nurses. Therefore, hospital administrators and nurse managers should pay more attention on this severe problem and take effective measures to alleviate burnout among nurses. According to the findings of this study, routine assessment of burnout among nurses should be added in health management strategies for nursing team.

In this study, job crafting was the most significant influencing factor for nurse burnout. Nurse managers should realize the positive effects of job crafting on nurse burnout. Several effective interventions (including job crafting training workshop, job crafting e-learning, and career crafting training) can be selected as training courses to meet the job crafting needs of nurses [58, 59]. Furthermore, nurse managers are encouraged to create a positive working environment and provide more organizational resources and opportunities for nurses to help them successfully deal with work challenges and achieve self-growth. In addition, several kinds of mutual-aid groups should be organized by nurse managers to support nurses find help from colleagues.

Leisure crafting is another new influencing factor for nurse burnout. According to this interesting finding, nurse managers are suggested to support nurses to cultivate personal hobbies and learn new knowledge via several group activities. Nurses are encouraged to fully use their leisure time to keep good personal relationships, develop new skills, and obtain a different experience. Setting achievable goals and keeping active thinking in leisure activities are also beneficial to nurses work attitude and performance.

## Figures and Tables

**Table 1:** Demographic characteristics and burnout among nurses (*n* = 1,235).

Characteristics	*n* (%)	Burnout (M ± SD)	Test statistics
*Sex*			
Men	52 (4.2)	1.98 ± 0.95	*t* = −0.271
Women	1183 (95.8)	2.02 ± 1.05	

*Age (years)*			
20–29	303 (24.5)	2.13 ± 1.05	*F* = 9.077^*∗∗*^
30–39	734 (59.4)	2.06 ± 1.03	
40–49	168 (13.6)	1.72 ± 1.05	
50–59	30 (2.4)	1.48 ± 0.97	

*Years of service*			
0–11	800 (64.8)	2.09 ± 1.03	*F* = 8.211^*∗∗*^
12–23	349 (28.3)	1.99 ± 1.06	
24–35	81 (6.6)	1.49 ± 0.96	
>35	5 (0.4)	1.93 ± 1.28	

*Academic degree*			
Diploma	8 (0.6)	1.51 ± 1.32	*F* = 1.569
Associate degree	144 (11.7)	2.09 ± 1.00	
Bachelor's degree	1050 (85.0)	2.01 ± 1.06	
Master's degree and higher	33 (2.7)	2.27 ± 0.78	

*Marriage*			
Single	209 (16.9)	2.29 ± 1.08	*F* = 6.002^*∗∗*^
Married	999 (80.9)	1.96 ± 1.03	
Divorced	26 (2.1)	2.05 ± 1.26	
Widowed	1 (0.1)	3.12 ± 0.00	

*Had child/children*			
No	294 (23.8)	2.26 ± 1.10	*t* = 4.470^*∗∗*^
Yes	941 (76.2)	1.95 ± 1.02	

*Employment*			
Temporary	696 (56.4)	2.07 ± 1.04	*F* = 2.649
Personnel agency	254 (20.6)	2.03 ± 0.99	
Authorized	285 (23.1)	1.90 ± 1.05	

*Professional qualification*			
Nurse	192 (15.5)	2.07 ± 0.96	*F* = 3.584^*∗∗*^
Senior nurse	412 (33.4)	2.11 ± 1.10	
Lead nurse	573 (46.4)	1.98 ± 1.03	
Associate chief nurse	50 (4.0)	1.74 ± 1.05	
Chief nurse	8 (0.6)	1.10 ± 0.66	

*Specialty area*			
Medical	402 (32.6)	2.10 ± 1.03	*F* = 2.592^*∗∗*^
Surgical	256 (20.7)	2.08 ± 1.03	
Gynaecology	106 (8.6)	1.86 ± 1.01	
Paediatric	89 (7.2)	2.26 ± 1.18	
Emergency	27 (2.2)	2.25 ± 1.01	
Operating room	55 (4.5)	1.89 ± 0.96	
Intensive care unit	53 (4.3)	1.92 ± 1.00	
Outpatient services	102 (8.3)	1.81 ± 1.05	
Others	145 (11.7)	1.86 ± 1.09	

*Monthly income^#^ (RMB, after tax)*			
≤3000	90 (7.3)	2.25 ± 1.16	*F* = 2.136
3001–5000	343 (27.8)	2.09 ± 1.06	
5001–7000	345 (27.9)	1.93 ± 1.02	
7001–9000	169 (13.7)	2.01 ± 1.03	
≥9001	288 (23.3)	1.99 ± 1.04	

*Shift work*			
No	370 (30.0)	1.88 ± 1.03	*F* = 5.643^*∗∗*^
<4 times/month	143 (11.6)	1.96 ± 1.01	
≥4 times/month	722 (58.5)	2.10 ± 1.06	

*Note*. ^*∗∗*^*p* < 0.01, ^#^1 US dollar = 6.54 Chinese Yuan.

**Table 2:** Descriptive and correlation analyses among burnout, job crafting, and leisure crafting of nurses.

	M ± SD	Min-max	Burnout	Job crafting	Leisure crafting
Burnout	2.02 ± 1.05	0.00–5.85	1		
Job crafting	4.05 ± 0.56	1.00–5.00	−0.400^*∗∗*^	1	
Leisure crafting	3.84 ± 0.78	1.00–5.00	−0.281^*∗∗*^	0.666^*∗∗*^	1

*Note*. ^*∗∗*^*p* < 0.01.

**Table 3:** K-means clustering analysis for burnout.

Groups	*n*	Final cluster centers	Scores
Low burnout group	678	1.24	0.00–2.10
High burnout group	557	2.97	2.11–5.85

**Table 4:** Model performance in predicting burnout in the validation cohort.

	AUC	Accuracy	Sensitivity	Specificity	Brier
Logistic	0.809	0.647	0.128	0.995	0.253
SVM	0.803	0.720	0.409	0.928	0.180
Random forest	0.820	0.733	0.443	0.928	0.167
Gradient boosting tree	0.821	0.739	0.470	0.919	0.161

## Data Availability

Data are available from the corresponding author upon reasonable request.

## Supplementary Materials

Supplementary Materials The supplementary file presents the detailed results of the binary logistic regression analysis, support vector machine (SVM), random forest, and gradient boosting tree.
