# Supplementary material for: Effects of Job Crafting and Leisure Crafting on Nurses' Burnout: A Machine Learning-Based Prediction Analysis
Source: J Nurs Manag. 2024 Jun 20;2024:9428519. doi: 10.1155/2024/9428519 (PMC11919151; doi:10.1155/2024/9428519)
Supplement: Supplementary Materials — The supplementary file presents the detailed results of the binary logistic regression analysis, support vector machine (SVM), random forest, and gradient boosting tree. [file 9428519.f1.docx]

**Supplementary file**

1. Participation rate and sample size

A total of 1,754 nurses from 122 care units in four tertiary hospitals were invited to complete the online questionnaires, and 1,235 responses were included in the final analysis, with response rates ranging from 65.8% - 90.41%. G*power 3.1.9.7 (Post hoc: compute achieved power) was employed to calculate the minimum sample size for the study. A 100% power was obtained, with a 0.15 effect size, a 5% margin of error, and a sample size of 1,235.

2. Results

eTable 1 and eFigure 1 present the results of Binary Logistic regression model. The Binary Logistic regression analysis showed that job crafting, age, medical department, paediatric department, and shift work significantly influenced nurse burnout. Nurses who were lower job crafting, younger age, worked in medical and paediatric departments and shifted work had a higher risk of experiencing severe burnout.

Support vector machine (SVM), random forest and gradient boosting tree were used to evaluate significant factors influencing nurse burnout. The importance of the permutation features was calculated using the three machine learning algorithms. In the SVM model, the top five predictors were job crafting, age, child/children status, years of service, and leisure crafting (Supplementary file eFigure 2). In the random forest and gradient boosting tree models , the top five predictors were job crafting, leisure crafting, age, years of service, and professional qualifications (Supplementary file eFigure 3 and eFigure 4).

eTable 1

Evaluation of the factors affecting nurse burnout with logistic regression model

| Determinants | Characteristics | β | SE | Z | OR | p |
| --- | --- | --- | --- | --- | --- | --- |
| Job crafting |  | -2.098 | 0.196 | 114.995 | 0.123 | <0.001 |
| Leisure crafting |  | -0.072 | 0.116 | 0.387 | 0.930 | 0.534 |
| Age |  | -0.439 | 0.173 | 6.445 | 0.645 | 0.011 |
| Years of service |  | -0.282 | 0.165 | 2.897 | 0.755 | 0.089 |
| Marriage |  | -0.057 | 0.229 | 0.061 | 0.945 | 0.805 |
| Had child/children |  | -0.225 | 0.233 | 0.930 | 0.799 | 0.335 |
| Professional qualification |  | 0.099 | 0.123 | 0.650 | 1.104 | 0.420 |
| Medical | No (reference) |  |  |  |  |  |
|  | Yes | 0.490 | 0.244 | 4.047 | 1.632 | 0.044 |
| Surgical | No (reference) |  |  |  |  |  |
|  | Yes | 0.358 | 0.261 | 1.883 | 1.430 | 0.170 |
| Gynaecology | No (reference) |  |  |  |  |  |
|  | Yes | 0.490 | 0.309 | 2.521 | 1.633 | 0.112 |
| Paediatric | No (reference) |  |  |  |  |  |
|  | Yes | 0.647 | 0.325 | 3.954 | 1.910 | 0.047 |
| Emergency | No (reference) |  |  |  |  |  |
|  | Yes | 0.535 | 0.472 | 1.284 | 1.708 | 0.257 |
| Operating room | No (reference) |  |  |  |  |  |
|  | Yes | 0.261 | 0.388 | 0.451 | 1.298 | 0.502 |
| Intensive care unit | No (reference) |  |  |  |  |  |
|  | Yes | 0.469 | 0.388 | 1.455 | 1.598 | 0.228 |
| Outpatient services | No (reference) |  |  |  |  |  |
|  | Yes | 0.346 | 0.319 | 1.178 | 1.414 | 0.278 |
| Shift work |  | -0.181 | 0.086 | 4.454 | 0.834 | 0.035 |
| Constant |  | 8.931 | 0.781 | 130.665 | 7565.693 | <0.001 |


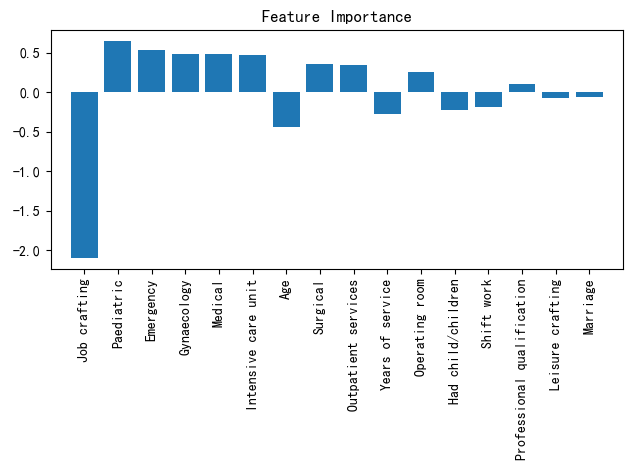


eFigure 1 The order of importance of influencing factors for burnout with logistic regression analysis


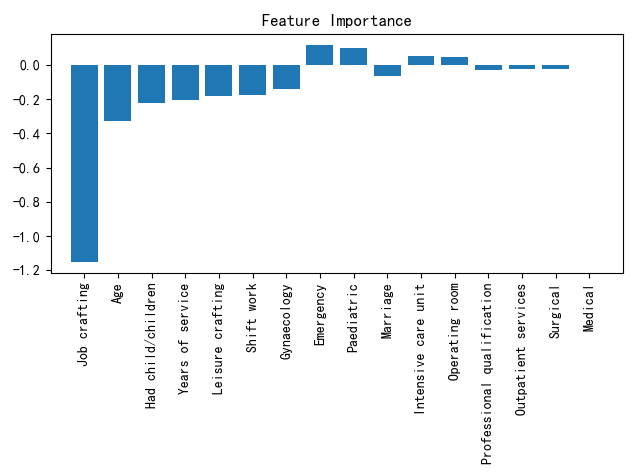
 eFigure 2 The order of importance of influencing factors for burnout with support vector machine


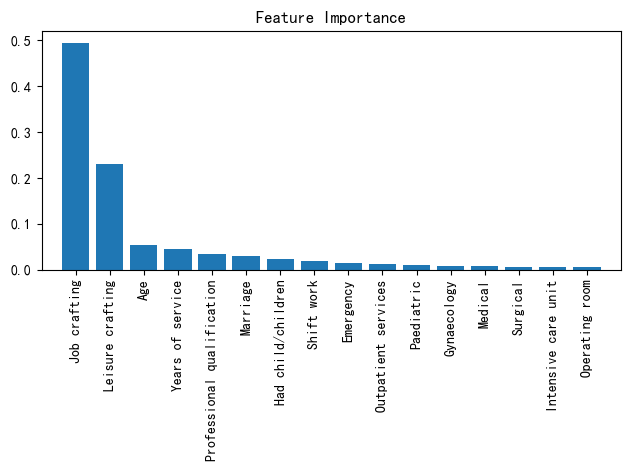


eFigure 3 The order of importance of influencing factors for burnout with random forest


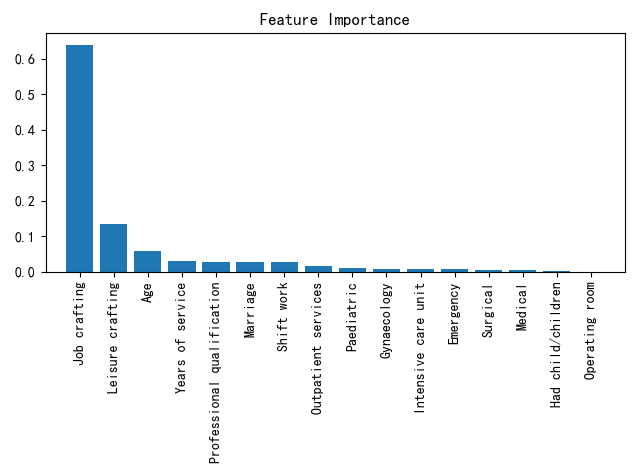


eFigure 4 The order of importance of influencing factors for burnout with gradient boosting tree
